# Quantitative Trait Locus Analysis of Leaf Morphology Indicates Conserved Shape Loci in Grapevine

**DOI:** 10.3389/fpls.2019.01373

**Published:** 2019-11-15

**Authors:** Elizabeth M. Demmings, Brigette R. Williams, Cheng-Ruei Lee, Paola Barba, Shanshan Yang, Chin-Feng Hwang, Bruce I. Reisch, Daniel H. Chitwood, Jason P. Londo

**Affiliations:** ^1^Department of Food Science, Cornell University, Geneva, NY, United States; ^2^Horticulture Section, School of Integrative Plant Science, Cornell Agritech at the New York State Agricultural Experiment Station, Geneva, NY, United States; ^3^State Fruit Experiment Station at Mountain Grove Campus, Darr College of Agriculture, Missouri State University, Mountain Grove, MO, United States; ^4^Institute of Ecology and Evolutionary Biology and Institute of Plant Biology, National Taiwan University, Taipei City, Taiwan; ^5^Instituto de Investigaciones Agropecuarias, INIA La Platina, Santiago, Chile; ^6^Bioinformatics Core, Knowledge Enterprise Development, Arizona State University, Tempe, AZ, United States; ^7^Department of Horticulture, Michigan State University, Lansing, MI, United States; ^8^Department of Computational Mathematics, Science and Engineering, Michigan State University, Lansing, MI, United States; ^9^Grape Genetics Research Unit, USDA-ARS, Geneva, NY, United States

**Keywords:** grapevine *(Vitis)*, leaf morphology, multivariate least squares interval mapping, phenotyping, quantitative trait loci

## Abstract

Leaf shape in plants plays important roles in water use, canopy structure, and physiological tolerances to abiotic stresses; all important traits for the future development and sustainability of grapevine cultivation. Historically, researchers have used ampelography, the study of leaf shape in grapevines, to differentiate *Vitis* species and cultivars based on finite leaf attributes. However, ampelographic measurements have limitations and new methods for quantifying shape are now available. We paired an analysis of finite trait attributes with a 17-point landmark survey and generalized Procrustes analysis (GPA) to reconstruct grapevine leaves digitally from five interspecific hybrid mapping families. Using the reconstructed leaves, we performed three types of quantitative trait loci (QTL) analyses to determine the genetic architecture that defines leaf shape. In the first analysis, we compared several important ampelographic measurements as finite trait QTL. In the second and third analyses, we identified significant shape variation *via* principal components analysis (PCA) and using a multivariate least squares interval mapping (MLSIM) approach. In total, we identified 271 significant QTL across the three measures of leaf shape and identified specific QTL hotspots in the grape genome which appear to drive major aspects of grapevine leaf shape.

## Introduction

Ampelography, the field of botany that uses leaf shape characteristics to classify and identify specific *Vitis vinifera* cultivars from one another, is built on the integration of a series of finite trait measurements (OIV, 2018); e.g., length of the midvein, length of the distal and proximal veins, depth of the sinus, and width of the petiolar gap. The culmination of all these measurements helps a researcher to distinguish between varieties. Although grapevine displays high phenotypic plasticity, these traits are rigorous enough to identify varieties, suggesting that the genetic architecture of this trait is heritable despite environmental influence. But how much of leaf shape is captured in measurements of these finite traits? Leaf shape is inherently quantitative, likely representing the complex interplay of many different genes and gene regulatory networks. Recent studies of leaf shape, and grapevine leaf shape in particular (Chitwood et al., 2014a; Chitwood et al., 2014b, Chitwood et al., 2016a, Chitwood et al., 2016b; Chitwood and Sinha, 2016; Chitwood and Otoni, 2017; Klein et al., 2017), have given rise to more sophisticated measures of leaf shape through landmarking and principal component analysis. Similarly, multivariate methods which decompose the complexity of shape into single value measurements may allow us to better capture the most important drivers of leaf shape.

In the age of genomics, it is possible to use these traits to map genetic loci impacting leaf shape. Leaf shape and form have been thought to contribute to plant speciation and survival through conferring adaptive advantages. As reviewed by different authors (Givnish, 1979; Givnish, 1987; Nicotra et al., 2011), plant leaf shape could affect temperature regulation, photosynthetic capacity, water use, and numerous other traits. A common theme regarding the ecophysiological importance of leaf shape is that leaf shape varies by climate. Evidence from fossilized leaves suggest that colder temperature climates select for leaves with larger and more abundant serrations as well as higher leaf dissection (Royer et al., 2005; Peppe et al., 2011). Interannual variation in leaf shape can be seen in grapevine, thus phenotypic plasticity is a critical aspect of realized shape (Chitwood et al., 2016b). Modern climates are predicted to generally warm as a result of climate change, placing increased pressure on grapevine leaves to tolerate heat stress. Heat stress effects on grapevine are typically associated with reduced fruit quality and degradation of anthocyanin compounds in the berry, but reduced photosynthesis and increased transpiration are also looming concerns for grapevine production (Greer and Weedon, 2013). Contrary to the trends of higher serration with cooler climates, leaf size and shape may also have large impacts on leaf temperature due to the rate of heat transfer across the boundary layer thickness on leaf surfaces. Heat dissipation from small leaves is faster than that from large leaves due to a reduced boundary layer and lobed leaves are predicted to also have faster heat transfer due to disruption of this layer. Lobed leaves may also have higher hydraulic efficiency due to their relative reduction in minor veins, which greatly increase the hydraulic resistance of the transpiration column (Sack and Holbrook, 2006; Nicotra et al., 2011). Understanding the genetic control of leaf shape patterning and the effects of different leaf shapes on grapevine productivity may contribute to our ability to continue to produce consistent fruit quality and yields despite climatic variation.

The only QTL analysis of leaf shape in grapevine to date was conducted using genetic maps developed with simple sequence repeat (SSR) markers in a population of the fungus-resistant (*Plasmopara viticola; Erysiphe necator*) *Vitis hybrid* “Regent” and the *V. vinifera* cultivar “Lemberger” (Welter et al., 2007). Fungal resistance and leaf morphology were evaluated as segregating traits in this population. For leaf morphology, 18 different finite traits (Galet, 1979; OIV, 2018) were mapped to 12 of 19 chromosomes. Substantial overlap of different trait QTL were observed on chromosome 1 and the inference was that these traits were related to sinus formation (Welter et al., 2007), or more generally, “lobiness”.

The objective of this study was to examine the genetic architecture of grapevine leaf shape through examining the shape of leaves from five different grapevine mapping families. We leveraged whole genome, genotyping-by-sequencing single nucleotide polymorphism (SNP) marker based genetic maps and three methods of evaluating leaf shape in order to investigate what regions of the genome contribute to leaf shape differences. Results demonstrate major and minor clusters of QTL in the grape genome that seem to partition based on prominent shape attributes, such as lobiness, as well as by species or genotype.

## Materials and Methods

### Plant Materials

**Figure 1:**
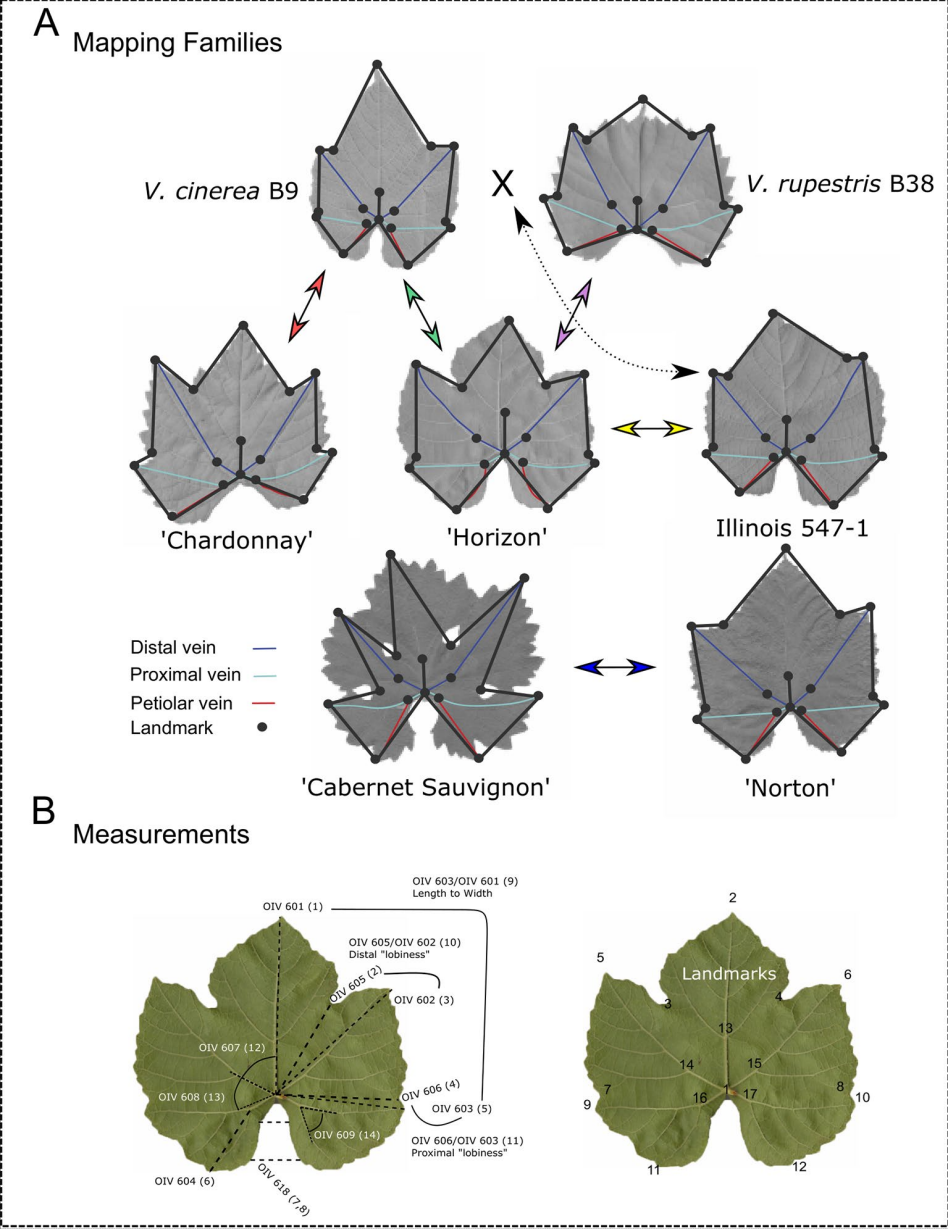
Landmark analysis and morphological features of parents from each mapping family. **(A)** Leaves representing morphologically diverse parents from each mapping family are displayed. Arrows between parents indicate crosses to produce mapping families. Dashed arrow indicates that Illinois 547-1 is itself a result of a cross between *Vitis cinerea* B9 and *Vitis rupestris* B38. The 17 landmarks (circles) and the distal (blue), proximal (teal), and petiolar (red) veins are marked on each parent. **(B) **OIV designations with corresponding finite traits measured in this study (in parentheses) and the 17 landmarks used to compute these measurements.

The five mapping families for this study were derived from the interspecific hybridization of diploid (2n = 38) *Vitis* species (**Table 1**; **Figure 1**). Four of the mapping families were grown at Cornell University on the Robbins Farm in Geneva, NY and included: 1) *V. vinifera* “Chardonnay” × *Vitis cinerea *B9; 2) “Horizon” (complex hybrid of *V. vinifera*, *Vitis labrusca* L., *Vitis rupestris* Scheele, and *Vitis aestivalis* Michx.) × *V. cinerea* B9; 3) “Horizon” × Illinois 547-1 (*V. rupestris* B38 × *V. cinerea* B9); and 4) *V. rupestris *B38 × “Horizon.” Six leaves were collected from the parents and all of the progeny from each of the four Cornell University mapping families. Leaves from each accession at developmental stages 5, 6, and 7 as defined by Chitwood et al. (2016a), were removed from the tip of two primary shoots exhibiting new vegetative growth (i.e. shoot tips with emerging leaves). Leaves were collected from secondary shoots if primary shoots were unavailable.

**Table 1:** Mapping families used in this study.

Parent 1 (female)	Parent 2 (male)	Family abbreviation	Number of progeny	Year crosses made	Location
“Chardonnay”	*Vitis cinerea* B9	CC	147	2009	Geneva, New York
“Horizon”	*Vitis cinerea *B9	HC	153	2009	Geneva, New York
“Horizon”	Illinois 547-1	HI	301	1988 and 1996	Geneva, New York
*Vitis rupestris* B38	“Horizon”	RH	205	2008	Geneva, New York
“Norton”	“Cabernet Sauvignon”	NCS	134	2005 and 2011	Mountain Grove, Missouri

The fifth mapping family, “Norton” (*V. aestivalis* hybrid) × *V. vinifera* “Cabernet Sauvignon” was grown in a research field at Missouri State University in Mountain Grove, MO. Four leaves were harvested from the parents and the progeny from the Missouri State University mapping family (**Table 1**). Leaves at developmental stages 6 and 7 were removed from two shoot tips.

During the field collection, leaves from each accession were placed directly into a Ziploc bag, labeled with a grapevine identification number, and stored in a cooler. All bagged leaves were either scanned on the same day (“Norton” × “Cabernet Sauvignon”) or placed in storage at 4°C until image procurement. Within 1 week of the field collection, the abaxial side of the leaves from each accession and the identification label were imaged using a scanner (Mustek A3 1200S; Mustek Systems, Hsinchu, Taiwan). Each image file was named with the grapevine identification number and an appended letter (if more than one scan was required to image all of the collected leaves from an accession).

### Collection of Shape Data

Fourteen different ampelographic measurements (OIV, 2018) were collected from the digital images (hereafter referred to as finite traits). Procrustes-adjusted coordinates were used in trigonometric functions (Euclid’s first book) to extract finite leaf attributes for QTL analysis (**Figure 1B**). The Pythagorean distance formula (*d*=*√*(*x*_2_-*x*_1_)^2^+(*y*_2_-*y*_1_)^2^) was used to determine the length of the midvein (OIV 601), the distance from the petiolar junction to the distal (OIV 605), and proximal (OIV 606) sinuses and to the distal and proximal lobe tips (OIV 602; OIV 603), and the length of the petiolar vein (OIV 604). The law of cosines (*b* ^2^=*a* ^2^+*c* ^2^-2*ac cos*(*B*)) was applied to obtain angles between the midrib and the distal vein (OIV 607), the distal and proximal veins (OIV 608) and the proximal and petiolar veins (OIV 609). The half width of the upper and lower petiolar gap (OIV 618) was obtained using the Pythagorean Theorem equation *b*=|*x*_2_-*x*_1_|. Ratios were also computed to capture rough estimates of leaf shape including length/width ratio (OIV 603/OIV 601), distal “lobiness” (OIV 605/OIV 602), and proximal “lobiness” (OIV 606/OIV 603).

Parental mean values for each family can be found in **Supplementary Table 1** and averaged measurements for each progeny genotype used in QTL analysis in **Supplementary Table 2**. Box plots were used to illustrate the phenotypic distribution of the traits measured (**Supplementary Figure 1A–K**).

### Landmark Analysis

Landmark analysis was performed as previously described by Chitwood et al. (2016a). Briefly, the ImageJ (Abramoff et al., 2004) point tool was used to plot 17 ordered landmarks as illustrated in **Figure 1B**. The ordered landmarks included the petiolar junction (1), the midvein tip (2), the left and right distal sinuses (3, 4), the left and right distal lobe tips (5, 6), the left and right proximal sinuses (7, 8), the left and right proximal lobe tips (9, 10), the left and right terminus petiolar veins (11, 12), the branch point midvein (13), the branch point left and right distal veins (14, 15), and the branch point left and right proximal veins (16, 17). To avoid multicollinearity in subsequent analyses, each leaf was examined as independent left and right halves, demarcated by the midrib.

### Morphometric Analysis and Data Transformation

**Figure 2:**
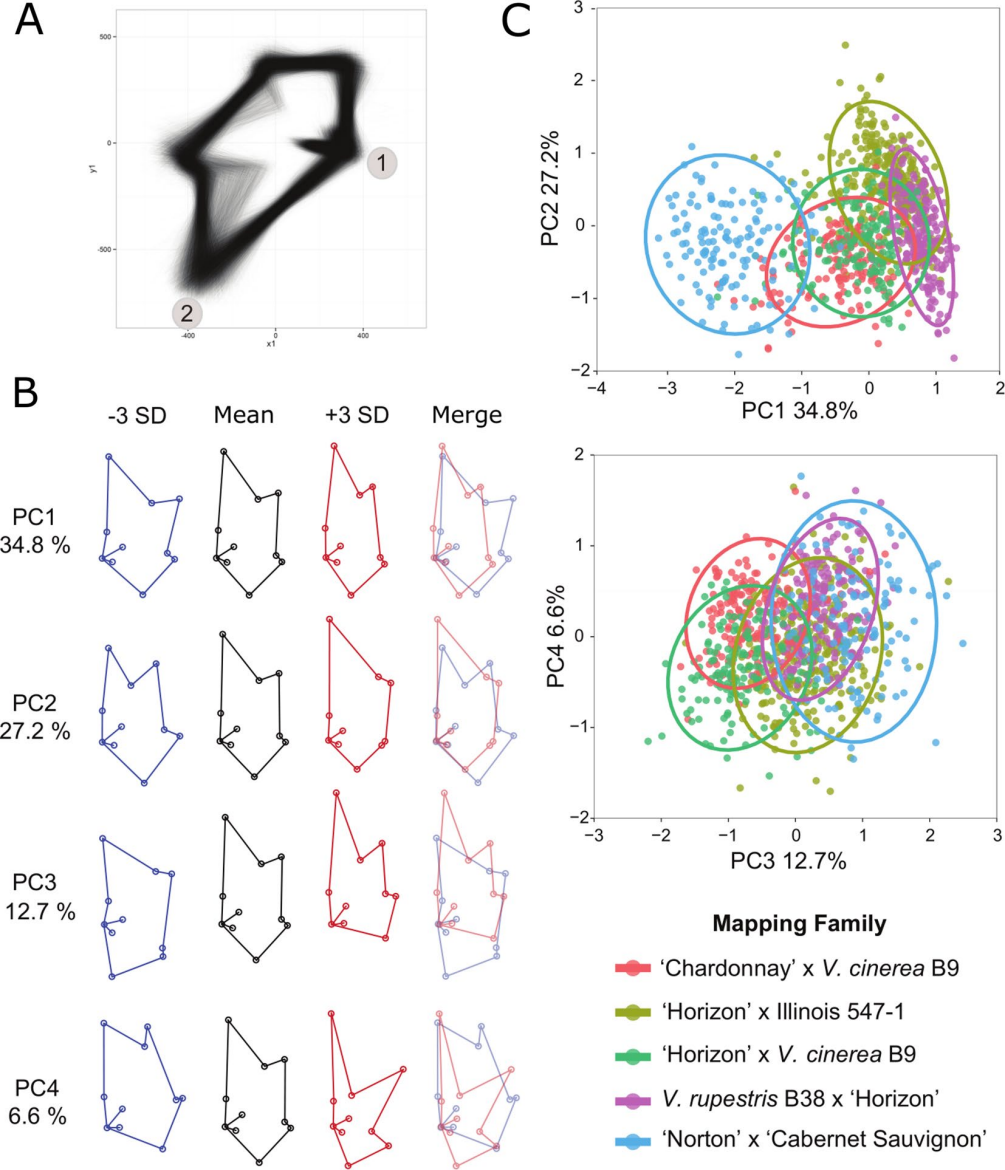
Principal component analysis (PCA) of Procrustes-adjusted coordinates. **(A)** Superimposed image of left and right leaf axes from the progeny of all five mapping families after generalized Procrustes analysis (GPA). Landmarks 1 and 2 are labeled for reference. **(B)** Eigenleaves from the top four principal components illustrate the variation of leaf shape within the five mapping families. The images represent the mean, +/− 3 standard deviations (SD; in blue and red, respectively), and the overlapping +/− SD. The percent of the phenotypic variation captured in each principal component (PC) is also listed. **(C)** Representative plots illustrate the relationships of the five mapping families using the PCs. Each family is represented by a different color as defined in the key and a 0.95 confidence interval ellipse.

A generalized Procrustes analysis (GPA) was performed on the landmark data set in R using the package shapes (Dryden and Mardia, 2016) and the procGPA function, reflect = TRUE. Reflection was allowed to superimpose the two halves of each leaf and Procrustes-adjusted coordinates were obtained from the procGPA object values. The final data set consisted of 10, two dimensional (x, y) coordinates for 10,780 leaf halves representing 5,390 whole leaves (**Supplementary Table 3**). The Procrustes-adjusted coordinates were graphed using ggplot2 (Wickham, 2009) in R (R Core Team, 2014) to create and assess a visual representation of each leaf half (**Figure 2**). Landmark and generalized Procrustes analyses were repeated to correct any confounded images identified during the visual screening.

The Procrustes-adjusted coordinates were further processed to analyze leaf shape as principal component (PC) scores and by using a multivariate approach. Landmark coordinates 14, 15, 16, and 17 were removed from the data set due to problems associated with multicollinearity. The Procrustes-adjusted coordinates from all five of the mapping families were pooled for the PCA so we could directly compare the PCs across each family. PC scores were obtained from the procGPA object values. The shapepca function was used to visualize eigenleaves (**Figure 2**). Trait values were averaged across each leaf half and genotype. The average trait value for the first four PCs were used in QTL analysis (**Supplementary Table 4**). Box plots were used to illustrate the distribution of the PCs for each family (**Supplementary Figure 2A–D**). After conducting the landmark analysis, the coordinates were divided into left and right sets along the midrib and the leaf shape data was transformed using GPA (**Supplementary Table 3**). A visual check was used to confirm proper placement of landmarks on each leaf replicate and proper reflection of the left and right leaf halves. Images from the visual check were superimposed to create a single image (**Figure 2A**).

Finally, the Procrustes-adjusted coordinates of points mirroring each other and leaf replicates for each accession were averaged for direct use in multivariate least squares interval mapping (MLSIM) (Anderson et al., 2011).

### Linkage Maps

Hyma et al. (2015) published the linkage maps for the *V. vinifera* “Chardonnay” × *V. cinerea *B9; 2) “Horizon” × *V. cinerea* B9; 3) “Horizon” × Illinois 547-1; and 4) *V. rupestris* B38 × “Horizon” using the 12X.0 PN40024 *V. vinifera* reference genome “PN40024” and the *de novo* pipeline. The map for “Norton” by “Cabernet Sauvignon” (Sapkota et al., 2019) was developed using the 12X.v2 PN40024 *V. vinifera* reference genome and the synteny pipeline as described in Hyma et al. (2015). For brevity, the five mapping populations will be referenced as abbreviations of the two parents of each population; CC, HC, HI, RH, and NCS (**Table 1**).

Linkage maps for the five mapping families were re-organized so that each cM represented a genetic marker in order to normalize genome coordinates for analyzing leaf shape using the multivariate approach. SNPs within the same cM were compiled into the same marker (since they mostly have identical genotype calls), and genotypes were imputed for cM positions without SNP data. To evaluate the phenotypic variance associated with the PCs of the entire leaf shape and the finite leaf attributes, the *jittermap* function from the R/QTL package was used to offset markers in identical positions in the linkage maps from each mapping family.

### QTL Analysis

The R/QTL package (Broman et al., 2003; Broman and Sen, 2009) was used to test for QTL associated with the finite leaf attributes and PCs of the entire leaf shape. Briefly, we calculated conditional genotype probabilities using *calc.genoprob*, step = 1, and default parameters. Next, we performed a genome scan using a single QTL model with the *scanone* function using a normal model, the Haley-Knott regression (Haley and Knott, 1992), and default parameters. We used 1,000 permutation tests to resolve logarithm of the odds (LOD) significance thresholds genome-wide for each parent. We used the *makeqtl* function to define inferred QTL and tested the significance of the model terms with the *fitqtl* function. Using the *refineqtl* function, we redefined QTL positions and again tested the significance of the model terms with the *fitqtl* function. Any non-significant QTL were removed. To search for additional QTL in the model, we used the *addqtl* function to add one QTL at a time to the model. Again, any non-significant QTL were removed, and the process repeated until a final QTL model was determined. The *addint* function was used to determine if any there were any pairwise interactions in the multiple QTL model and the *lodint* function was used to report a 1.5 LOD supported interval for each significant QTL. We calculated QTL effects as the difference in the mean phenotype value of individuals within each genotype class at the marker with the highest LOD score, using the *effectplot* function.

MLSIM, the multivariate counterpart of composite interval mapping (Anderson et al., 2011), was used to analyze the entire leaf shape with the averaged Procrustes-adjusted coordinates of points mirroring each other. First, multivariate analysis of variance (MANOVA) was applied to all markers across the genome to identify the marker with the strongest effect. To identify the second strongest QTL, MANOVA was again performed across all markers, using the first QTL as a cofactor. The process was repeated until no further significant QTL were identified. Statistical significance was determined by comparing the true statistic (i.e. Wilks’ lambda) to those from 1,000 permuted datasets, where the genotype-phenotype relationship was randomized across all individuals. To further refine the list of significant QTL, we calculated a rough estimate of the percent variance based on the true statistic using 5% as an arbitrary cutoff to define phenotypic significance.

### Identification of Candidate Genes

For QTL regions where multiple shape QTL overlapped and the traits tied to those QTL were similar across mapping families, annotated genes were reviewed for potential candidate gene identification. Using overlapping intervals for the various QTL, gene identities were extracted from the grapevine VCOST.V3 gene annotation (https://urgi.versailles.inra.fr/Species/Vitis/Annotations) and gene identities were cross referenced with the V1 functional annotation (Grimplet et al., 2009, Grimplet et al., 2012) and CRIBI grape database (Vitulo et al., 2014). Identities of candidate genes are listed in the following format; candidate gene (V3 gene name; V1 annotation) (**Supplementary Table 8**).

## Results

### Leaf Morphometrics and Quantitative Trait Loci Analysis of Five *Vitis* Mapping Families

Each parent from the five mapping families has unique shape attributes as illustrated in **Figure 1A**. For example, *V. rupestris* B38 has a reniform leaf shape, whereas “Chardonnay” has an orbicular leaf shape. Differences in lobing patterns and the petiolar sinus can also be observed across the parents. Box plots depict the phenotypic distribution of each finite trait attribute within each of the five mapping families in **Supplementary Figure 1**, and comparisons of finite trait measurements for each family are presented in **Supplementary Table 1**. In general, *V. cinerea* has the longest leaves, *V. rupestris* has the shortest. “Cabernet Sauvignon” has the most lobed shape of all the parents while Illinois 547-1 and *V. cinerea* are the least lobed. *V. rupestris* has the largest petiolar gap of all the other parents while *V. cinerea* has the smallest. Because the relative differences in these finite traits between parents shifts when comparing between families (e.g. “Horizon” is the “thinner” parent in the *V. rupestris* family, but is the “wider” parent in the *V. cinerea* family), finite trait QTL do not always map to the same genomic location or may be only identified from a subset of the families tested.

**Figure 3:**
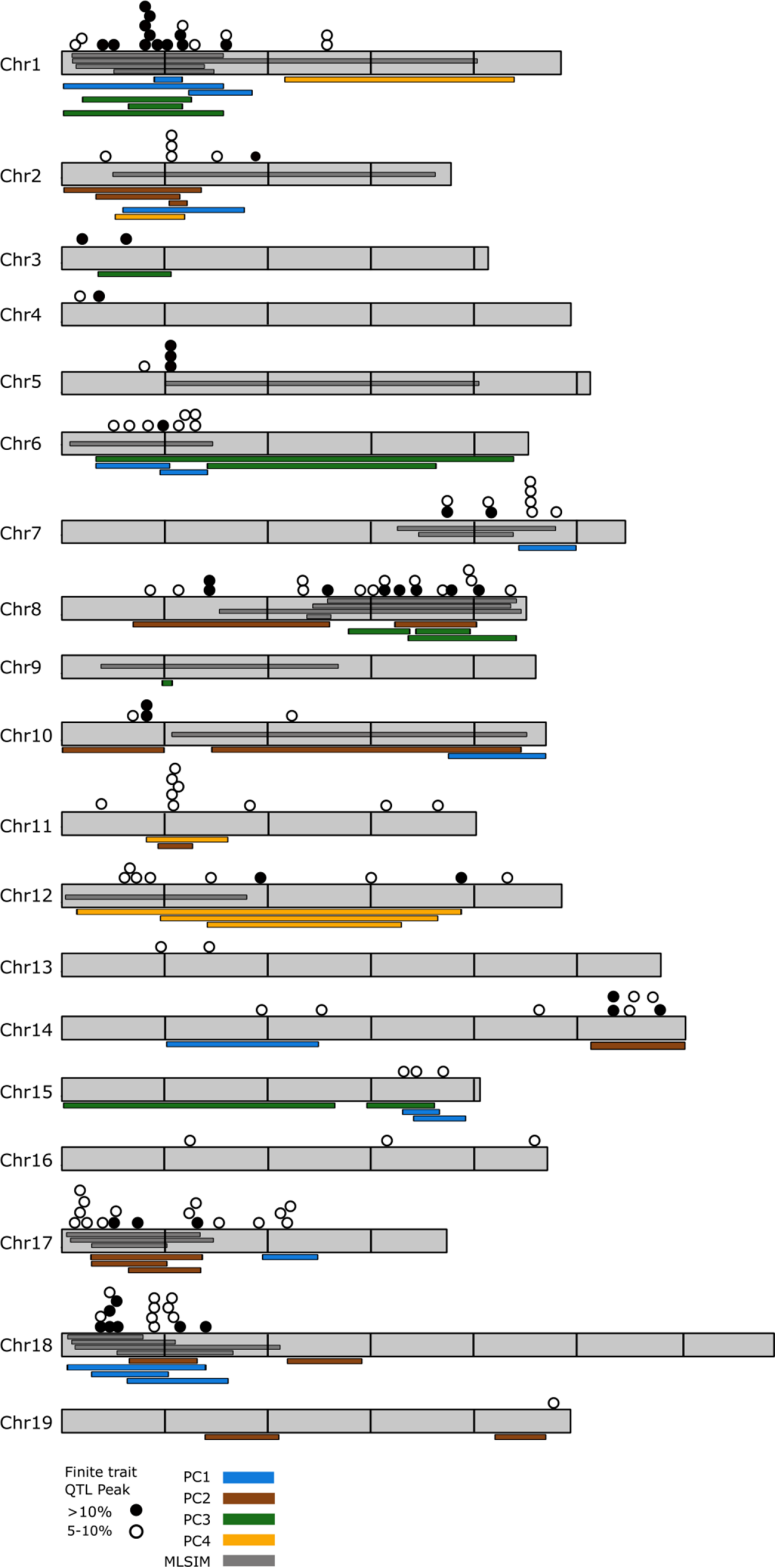
Genome-wide distribution of observed quantitative trait loci (QTL) for shape traits. Overlay of all QTL that explain greater than 5% of variation that were observed in this study. Chromosomes are shown in gray and are scaled based on the PN20024 *Vitis vinifera* reference genome with horizontal lines marking 5M bp increments. Filled circles represent QTL that explain greater than 10% shape variation for OIV ampelographic traits; empty circles represent QTL that explain between 5 and 10% variation. Colored bars adjacent to the chromosomes indicate QTL detected using principle coordinate analysis. Multiple bars of the same color that overlap indicate PC QTL detected in multiple mapping families. Dark gray bars depicted within the chromosomes indicate QTL detected using multivariate least squares interval mapping (MLSIM) methods. Multiple bars that overlap indicate MLSIM QTL detected in multiple mapping families.

QTL analysis of the finite trait/OIV leaf measurements identified QTL on every chromosome except chromosome 9 (**Supplementary Table 5**). There were 147 significant QTL for finite traits when considering all loci that explain greater than 5% variation for any single trait. Despite visually obvious differences between parents regarding aspects of lobe size in the NCS population, reduced leaf sampling in this family diminished our ability to identify significant QTL for many of the finite traits in this family. Forty-nine QTL explained 10% or greater variation. Clusters of 3 or more QTL were noted on chromosomes 1, 2, 5, 6, 7, 8, 10, 11, 12, 14, 15, 17, and 18 (**Figure 3**).

Results of the PCA analysis demonstrated that the first four PCs accounted for 81.3% of the total phenotypic variance in the 5 mapping families (**Figure 2**) and resolved 47 QTL, each explaining at least 5% of phenotypic variation (**Supplementary Tables 4** **,** **6**). The mean shape with +/− 3 standard deviations for PCs one through four are depicted in **Figure 2**. Box plots illustrate the distribution of each PC within each of the five mapping families (**Supplementary Figure 2**). Pairwise comparisons of the PC’s (**Figure 2**) demonstrated that PC1 and PC2 separate primarily based on mapping family parentage. The most distantly related family, NCS, is an outlier from the other four mapping families. The two families that share *V. cinerea* B9 as a parent cluster together, whereas the family that has *V. rupestris* B38 as a parent is separated and the mapping family HI fills in the gap between the families.

QTL analysis of the leaf shape as a multivariate trait using MLSIM explained between 83.12–87.98% of variation between the mapping populations and resolved 23 significant QTL that explain greater than 5% of phenotypic variance (**Supplementary Table 7**). Circular plots, constructed using the program Circos (Krzywinski et al., 2009), illustrate overlap between PC and MLSIM QTL by family and by parental origin and can be found in **Supplementary Figures 3** and **4**.

Combining the three methods for assessing leaf shape in grapevine resolved 217 significant QTL that explain at least >5% of phenotypic variation. Genomic regions where at least three or more QTL components were observed on many chromosomes and can be broken into major and minor clusters (**Figure 3**). Major clusters, those which occur between finite QTL of >10% and PCA and/or MLSIM QTL occur on chromosomes 1, 8, 17, and 18 (**Figure 3**). No major clusters between finite traits and PC4 loci were detected. Minor clusters, those which occur between finite QTL of 5–10% and PCA or MLSIM QTL occur on chromosome 2, 5, 7, 10, 11, 12, 14, and 15.

The major QTL clusters on chromosomes 1, 8, 17, and 18 were evaluated for potential candidate genes related to aspects of leaf shape and organ development. The reference annotation for the 7.35 Mb QTL region of chromosome 1 (93,586–7,453,254) indicated 813 predicted grapevine genes for which 530 have annotated function. On chromosome 8 we examined an 8.16 Mb region (13,902,173–22,064,043) which contains 1,101 predicted genes, 777 with annotated functions. The QTL overlap region of chromosome 17 spanned a 5.5 Mb region (1,321,665–6,831,362) containing 548 genes, 348 with annotated function. The final major overlap region on chromosome 18 spanned 7.79 Mb (274,922–8,070,602) and contained 858 genes, 573 with annotated function.

## Discussion

What is the genetic architecture of something as complex and quantitative as leaf shape? In designing this analysis, we were most interested in understanding if there were conserved genomic regions that set the underlying complexity of leaf shape in *Vitis.* Welter et al. (2007) previously explored this concept using SSR markers in a single mapping family and identified chromosome 1 as a major player in determining leaf sinus depth. However, this study was conducted within a very limited genetic background. In our study we combined leaf shape measurements across five different families. We could have chosen to conduct five different QTL studies using the finite ampelographic traits as well as the PC and MSLIM methods described above. However, we quickly realized that PC and MSLIM based QTL would change between mapping families, as the morphospace defined by each family is unique in important ways. This can be seen by comparing how parental means contrast in the families which include “Horizon”. Sometimes “Horizon” represents the parent with larger leaf measures, sometimes smaller (**Supplementary Table 1**). As such, PC1 in each family alone would not be comparable to PC1 in any other family. Thus, in this analysis we chose to examine grapevine leaf shape as it exists within a larger grape leaf morphospace, defined by all five mapping families. As such, we performed our finite trait, PC, and MSLIM analysis all within the same coordinate plane. By doing so, we resolved that there are a few major chromosomal loci that play a role in determining grapevine leaf shape. As a tradeoff to this approach, we lose some resolution to define clear differences in leaf shape within each family. For example, the lower sampling of leaves in the NCS family reduced our ability to detect QTL, despite clear visible differences in leaf shape between these two parents.

While humans are inherently good at discerning shape as “different”, it is often very challenging to describe the specific aspects of shape that differ between two complex objects, like leaves. For example, when comparing the morphology of “Cabernet Sauvignon” with that of *V. cinerea*, it is very easy to see distinct differences in sinus depth/lobiness. PCA analysis and multivariate analysis demonstrated that there are significant differences in shape, but they are challenging to explain what specific parts of shape are driving those differences. In this study we combined analysis of various aspects of grapevine leaf shape, starting with traditional ampelographic measurements and finishing with multivariate analyses of the entirety of leaf shape as a single measure. We observed 217 significant QTL in this study spread across every chromosome of the grapevine genome, demonstrating that leaf shape in grape is influenced by many different genes and allelic combinations (**Figure 3**). Some chromosomes, like chromosomes 4, 9, 13, 14, 16, and 19 had relatively very few loci. In contrast, hotspots of QTL based on the different shape measurements were found, particularly on chromosomes 1, 8, 17, and 18.

### Major Quantitative Trait Loci Clusters

Chromosome 1 represents a major QTL cluster for leaf shape and this QTL cluster overlaps with the major leaf shape QTL detected in a previous study of a mapping family made between two *V. vinifera* cultivars, “Lemberger” and “Regent” (Welter et al., 2007). In that study, linkage group 1 held QTL for OIV 606 (proximal sinus depth). Our finite trait QTL analysis also indicated loci for proximal (OIV 606) and distal sinus depth (OIV 605), as well as the angle between the proximal and petiolar veins (OIV 609) in the HC (14%; 27%; 17% of variation explained, respectively) family; and for proximal lobe width (OIV 603) in the HI (12%) family. Most interestingly, QTL across families were detected for distal lobiness (OIV 605/OIV 602) in the HC (22%), HI (12%), and RH (18%) families with the contributing parent being “Horizon”. Similarly, proximal lobiness (OIV 606/OIV 603) was detected in the CC (13%), HC (23%), HI (11%), and RH (15%) families with the contributing parent being “Horizon” for the HC, HI, and RH families and “Chardonnay” in the CC family. These clusters of finite traits also overlap with QTL for PC1 suggesting that the major shape component in PC1 is lobiness. PC1 QTL were observed in the HC and HI families with the QTL observed in the PC1 QTL explained 28% of the variation in the HC family while also explaining 9 and 8% of the variation in the HI family (coming from each parent). PC3 QTL were also seen to overlap with these finite traits on chromosome 1 and explained variation in three families; CC (11%), HC (25%), and HI (6%). MLSIM QTL clustered on chromosome 1 were observed in the HC, HI, and RH families. This QTL was the MLSIM QTL which explained the majority of the shape variation for both the HC and RH families, explaining 47 and 41% of leaf shape respectively, also suggesting that lobiness is a major defining characteristic in these families. The two remaining QTL in the HI family describe 13 and 22% of variation (35% total) but the QTL are contributed each by a single parent. Taken together, the chromosome 1 QTL cluster appears to primarily describe differences in the lobiness of leaves and may suggest that the genetic architecture that determines distal lobiness are in part shared by those which determine proximal lobiness.

The resolution of the QTL on chromosome 1 differs by mapping family but the QTL peak and intervals for distal and proximal lobiness consistently overlap across “Horizon” based families (**Supplementary Table 5**). Of the many candidate genes in this QTL region, several stand out as having previously been shown to affect leaf morphology. The first is a *Vitis* ortholog of the JAGGED gene (Vitvi01g01939; VIT_01s0011g03600), a gene that has been shown to encode a zinc finger protein promoting leaf tissue development, specifically affecting leaf serrations in *Arabidopsis* (Dinneny et al., 2004; Xu et al., 2008). JAGGED genes are related to the LATERAL ORGAN BOUNDARIES (LOB) domain genes ASYMMETRIC LEAVES (AS1 and AS2; (Xu et al., 2008). This gene is found near the edge of the QTL interval and two additional LOB related genes can be found just outside this region. The developmental gene Wuschel related homeobox 1, VvWOX1 (Vitvi01g00427; VIT_01s0011g05020), is also found in this region. WOX1 has been implicated in embryogenesis and lateral organ formation as well as cultivar specific expression in grapevine (Boccacci et al., 2017). It is interesting to note that these two candidate genes, JAGGED and WOX1, were also identified as laying between the significant SNPS associated with differences in leaf shape as detected by a GWAS based study of the USDA cultivated grapevine repository (Chitwood et al., 2014b). A third potential candidate gene is the DELLA family GRAS transcription factor VviRGA5 (VviRGA5; VIT_01s0011g05260). DELLA family members play a role in gibberellin signaling as well as stress response (Grimplet et al., 2016). Grapevine heteroblastic leaf shape differences as they emerge from the base of the cane to the apex (Chitwood et al., 2016a; Chitwood et al., 2016b), as well as the potential role of GA in tendril *versus* inflorescence determination (Boss and Thomas, 2002; Díaz-Riquelme et al., 2014) supports the hypothesis that GA may play a role in leaf shape differences in grapevine.

The major cluster of loci on chromosome 8 describes a set of QTL for finite traits within single mapping families but their physical distribution is much more diverse than that observed on chromosome 1. QTL for midvein length (OIV 601) and the angle between the proximal and petiolar veins (OIV 609) were observed in the HC family (11%; 15%), the length of the distal vein (OIV 602) in the NCS family (10%), and the length of the petiolar vein (OIV 604) in the HR family (13%). Two traits had QTL detected in multiple families; depth of the proximal lobe (OIV 603) in the CC and HR families (11%; 13%), and the ratio of lobe and sinus for proximal lobiness (OIV 606/OIV 603) in the CC (13%), HC (10%), and HR (13%), suggesting there are secondary loci for lobiness. In general, this QTL cluster seemed to have more QTL associated with proximal lobe traits than that of the cluster on chromosome 1 and in the CC and HC families, these lobe QTL are coming from the *V. cinerea* parent. Overlap of finite traits occurs on chromosome 8 with QTL for PC2 and PC3 as well as MLSIM QTL. PC2 QTL are observed from the CC (17%) and HC (7%) families but only the HC QTL overlaps with the rest of the cluster. PC3 QTL are observed in the CC (15%), RH (17%), and NCS (13%) families. MLSIM QTL that overlap in this region originate in the CC (13%), HC (23%), RH (21%), and NCS (38%) families. This MLSIM QTL explained the greatest percent shape variation for the NCS family, perhaps capturing aspects of *V. aestivalis* ancestry. Among the many potential candidate genes in this region is the *Vitis* ortholog of the BLADE-ON-PETIOLE2 (BOP2) gene (Vitvi08g01678;VIT_08s0007g05740), a gene that interacts with KNOX genes and controls leaf morphogenesis, patterning, and heteroblasty in *Arabidopsis* (Ha et al., 2003; Ha et al., 2007) and impacts development of the leaf blade in rice (Toriba et al., 2019). In *Arabidopsis*, BLADE-ON-PETIOLE (BOP) genes have been shown to play a role in regulating LOB genes, similar to the JAGGED genes mentioned above on chromosome 1.

The major cluster of loci on chromosome 17 includes a lower number of QTL that explain >10% of variation in finite traits compared with the major clusters on chromosomes 1, 8, or 18. However, there is a large concentration of loci explaining between 5 and 10%. The three loci that explain >10% of variation describe the trait of petiolar gap width (OIV 618) in the RH (13%) family as well as the size of the distal vein (OIV 602) in the HC (14%) and HI (12%) families. The QTL observed from PCA analysis that overlap in this region describe PC2 for the HC (7%), HI (6%), and NCS (16%) families. This is the PCA based QTL which explains the greatest amount of shape variation in the NCS family. The three overlapping MLSIM QTL in this region are all relatively minor, relative to percent variation explained, and come from the HC (12%), HI (6%), and RH (8%) families. This loci appears to be associated with shape expression in the “Horizon” genetic background, as all the finite, PC, and MLSIM QTL identified are in the three families where this parent is represented. “Horizon” is a complex hybrid cultivar with ancestry stemming from *V. vinifera, V. labrusca,* and *V. rupestris*. The finite trait QTL in this cluster are widely distributed across the PC and MLSIM intervals and don’t describe consistent traits across families. However, the region does have several annotated zinc finger proteins as well as a Wuschel-related homeobox protein, it is difficult to make many generalizations of which specific shape that this cluster contributes.

The final major cluster of loci occurs on chromosome 18 and represents overlap of finite traits, PC1, and MLSIM loci. The finite traits that occur in this cluster occur as single family and multi-family traits. Instances that occur as single-family QTL describe length of the distal vein (OIV 602) for the HC (9%) family, length of the proximal vein (OIV 603) for the HI (6%) family, and distal lobiness (OIV 605/OIV 602) for the CC (15%) family. Overlap of two families occurred for petiolar gap (OIV 618) in the HI (6%) and RH (6%) families, as well as for the angle between the mid and distal vein (OIV 607) for the HC (14%) and HI (9%) families. Two traits were observed in three families; distance to the distal sinus (OIV 605) in the CC (15%), HC (8%), and HI (5%) families, and length of petiolar vein (OIV 604) in the HC (14%), HI (8%), and NCS (11%) families. Overlap of PC1 occurs in this region in the CC (14%), and HI (9%, 8%) families with both parents contributing in the HI family. Of the two PC2 loci on chromosome 18, only one overlaps with the finite trait cluster and this locus is coming from the HC (9%) family. Finally, MLSIM that overlap in this region include the major QTL for the CC (38%) family, second greatest QTL for the NCS (23%) family and minor QTL for the HC (5%), and RH (11%) families. It is interesting that “Horizon” based loci are less represented in this cluster. In the CC and NCS families, the QTL variation can be attributed to the “Chardonnay” and “Cabernet Sauvignon” parents and suggests that the QTL cluster on chromosome 18 is more associated with the shape of *V. vinifera* cultivars than wild grapevine species or those with hybrid ancestry. The only overlap in finite trait at this locus is the sinus depth (OIV 605) and distal lobiness (OIV 605/602) from “Chardonnay”. A survey of the annotated genes in this region reveals two candidate genes that have been linked to development and leaf shape function, a Wuschel-related homeobox 13 gene, WOX13 (Vitvi18g00084; VIT_18s0122g0114) and cup-shaped cotyledon3, CUC3 (Vitvi18g00052; VIT_18s0122g00800). WOX13 has been shown to impact cell fate determination in the moss model system *Physcomitrella patens* (Sakakibara et al., 2014) and plays a constitutive role in the patterning of most developing tissues in rice (Minh-Thu et al., 2018). In *Arabidopsis*, CUC3 seems to play a role in defining axillary meristems and boundary patterning of stems, pedicels, and leaves (Hibara et al., 2006) and silencing of this gene results in the loss of leaf serrations and leaf lobing in the compound leaves of *Passiflora*, *Pisum*, and *Cardamine* (Blein et al., 2008). As the locus on chromosome 18 seems to be tied more closely with “Chardonnay” and “Cabernet Sauvignon” this locus may act synergistically or in parallel to the one detected on chromosome 1.

What is the value in being able to select or breed for increased lobiness in grapevine? One potential benefit may be as a method for mitigating the effects of warming summer temperatures through increasing canopy air movement and through decreasing the leaf boundary layer (Nicotra et al., 2011). A more open canopy could allow for more sunlight to penetrate through and increase berry quality components (Crippen and Morrison, 1986; Morrison and Noble, 1990;Hunter et al., 1991). Additionally, increased airflow through the canopy could help reduce microclimate attributes that pathogens require. For example, reducing humidity may help fend off plant pathogens like downy mildew and black rot that rely on humid conditions (Spotts, 1977; Lalancette et al., 1988; Austin et al., 2011; Austin and Wilcox, 2012). Relatedly, a more open canopy may allow enhanced penetration of pesticide sprays (Travis et al., 1987). The genes underlying the major QTL on chromosomes 1, 8, and 18 may prove to be a way forward in producing and selecting for climate adapted grapevines and this work provides the markers and foundation to move to the next phase of fine mapping.

## Conclusion

The results of this study clearly demonstrate the complex genetic architecture associated with leaf shape in grapevine. While a huge number of QTL were identified, most explained low percentages of the phenotypic variation. When examined as a complex phenotype, we saw that these loci tended to cluster in specific genomic regions and suggest that chromosome 1 represents a major locus associated with perhaps the easiest shape characteristic to observe, lobiness. Several potential candidate genes underlie this major locus as well as the loci on chromosomes 8 and 18 and future studies with fine mapping may uncover the causal aspects of leaf lobing in grapevine. Future mapping efforts in populations with even greater leaf morphology differences may elucidate the key genes in the lobiness loci that could be targets for gene editing in existing grape cultivars. Our study helps lay the foundation needed for future marker assisted based breeding efforts for controlling aspects of leaf canopies in grapevines and other crop species. Selecting for designer leaves might be an important aspect in the development of new cultivars with idealized canopy structure.

## Data Availability Statement

The datasets generated for this study can be found in the NCBI SRA http://www.ncbi.nlm.nih.gov/bioproject/281110.

## Author Contributions

JL and DC contributed the conception and design of the study. PB and SY produced genetic maps. ED, JL, and BW conducted leaf landmark measurements. ED conducted QTL analyses. C-FH and BR produced mapping families. C-RL designed and conducted MLSIM analysis. ED and JL led the writing of this manuscript. All authors contributed to manuscript revision, and read and approved the submitted version.

## Funding

This work was supported by the U.S. Department of Agriculture-NIFA Specialty Crop Research Initiative grant number 2011-51181-30635, by the National Grape Research Alliance, Michigan State University AgBioResearch, Comisión Nacional de Investigación Científica y Tecnológica (CONICYT) CONICYT PFCHA/DOCTORADO BECAS CHILE/2011 - 72120446, and the Taiwan Ministry of Science and Technology grant number 108-2636-B-002-004. We thank Steve Luce and Mike Colizzi for Cornell research vineyard care and maintenance.

## Conflict of Interest

The authors declare that the research was conducted in the absence of any commercial or financial relationships that could be construed as a potential conflict of interest.
