# Supplementary material for: Quantitative Trait Locus Analysis of Leaf Morphology Indicates Conserved Shape Loci in Grapevine
Source: Front Plant Sci. 2019 Nov 15;10:1373. doi: 10.3389/fpls.2019.01373 (PMC6873345; doi:10.3389/fpls.2019.01373)
Supplement: Supplementary file 1 [file Image_1.pdf]

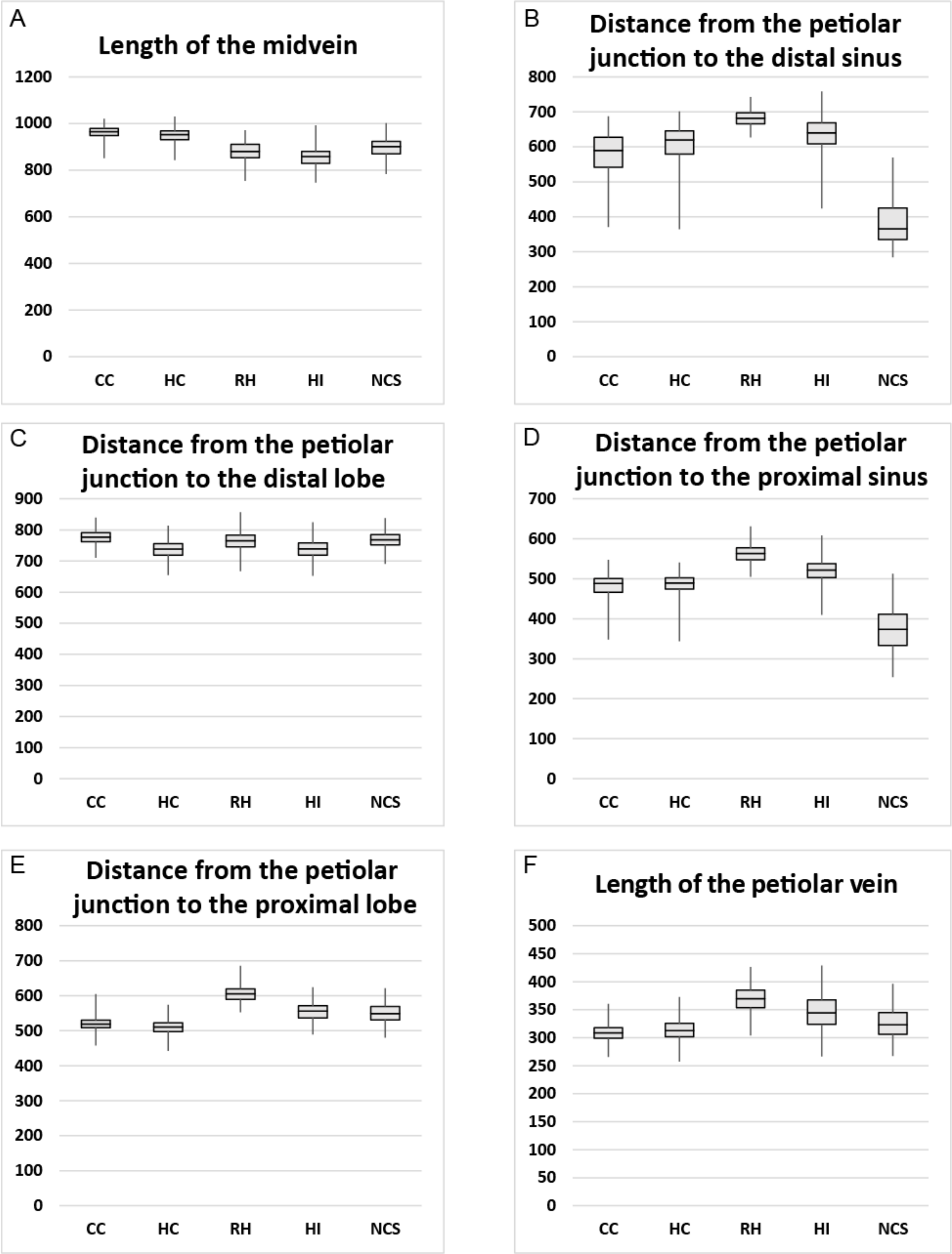
A
Length of the midvein
1200
1000
800
600
400
200
0
CC
HC
RH
HI
NCS
B
Distance from the petiolar junction to the distal sinus
800
700
600
500
400
300
200
100
0
CC
HC
RH
HI
NCS
C
Distance from the petiolar junction to the distal lobe
900
800
700
600
500
400
300
200
100
0
CC
HC
RH
HI
NCS
D
Distance from the petiolar junction to the proximal sinus
700
600
500
400
300
200
100
0
CC
HC
RH
HI
NCS
E
Distance from the petiolar junction to the proximal lobe
800
700
600
500
400
300
200
100
0
CC
HC
RH
HI
NCS
F
Length of the petiolar vein
500
450
400
350
300
250
200
150
100
50
0
CC
HC
RH
HI
NCS

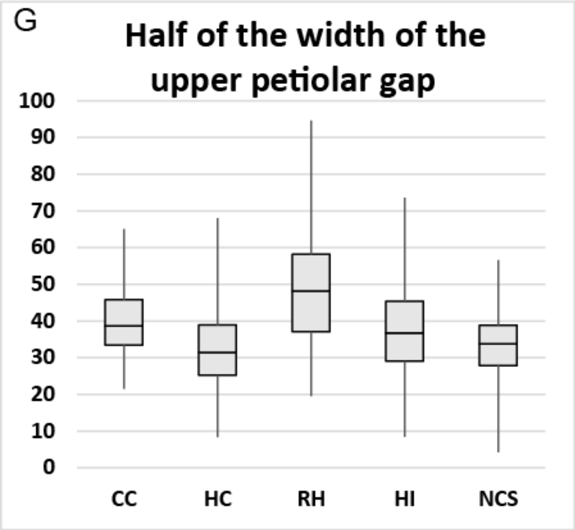

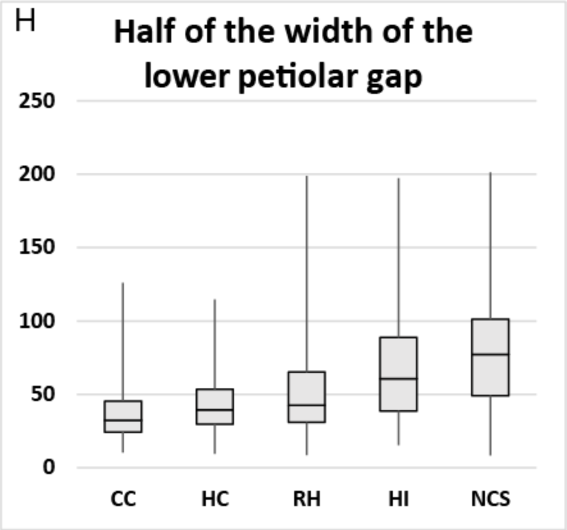

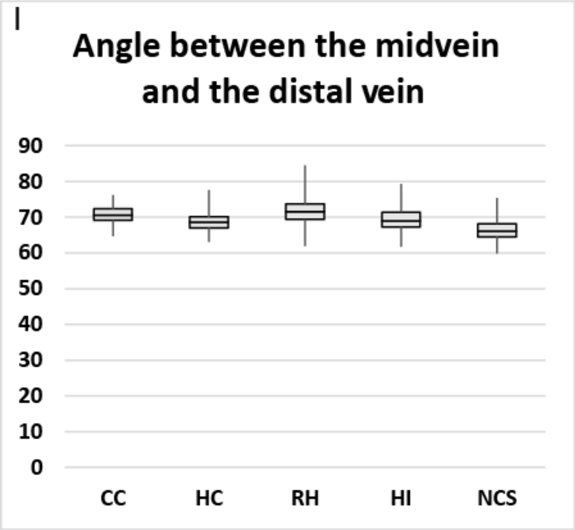

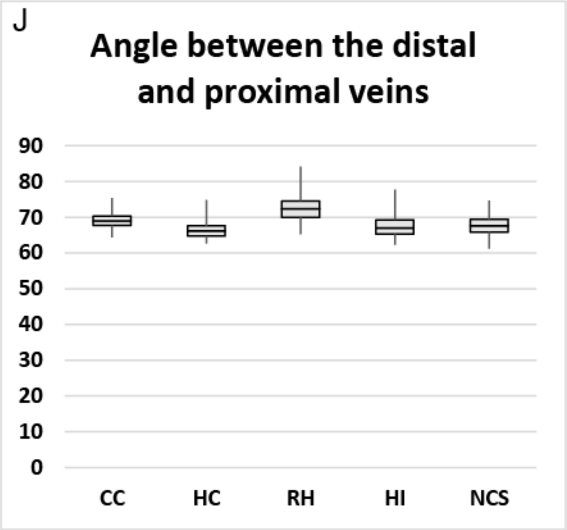

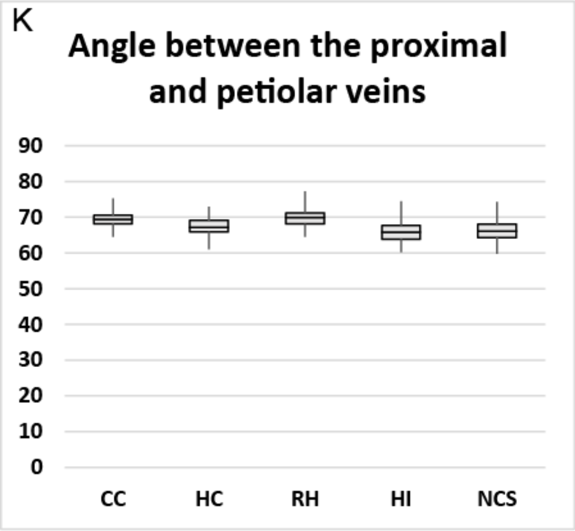

Supplementary Figure 1. Boxplots of ampelographic (OIV) finite trait data for each mapping family. Y-axis values represent comparative values derived from the Procrustes-adjusted coordinate space of all 5 families. The center line in each box represents the median value. The top box represents the first quartile of the data and the bottom box represents the third quartile of the data. The top and bottom whiskers represent the maximum and minimum values (respectively) in the dataset. CC: *V. cinerea* B9 by 'Chardonnay;' HC: 'Horizon' by *V. cinerea* B9; RH: *V. rupestris* B38 by 'Horizon;' HI: 'Horizon' by Illinois 547-1; and NCS: 'Norton' by 'Cabernet Sauvignon.'
