# Supplementary material for: Quantitative Trait Locus Analysis of Leaf Morphology Indicates Conserved Shape Loci in Grapevine
Source: Front Plant Sci. 2019 Nov 15;10:1373. doi: 10.3389/fpls.2019.01373 (PMC6873345; doi:10.3389/fpls.2019.01373)
Supplement: Supplementary file 2 [file Image_2.pdf]

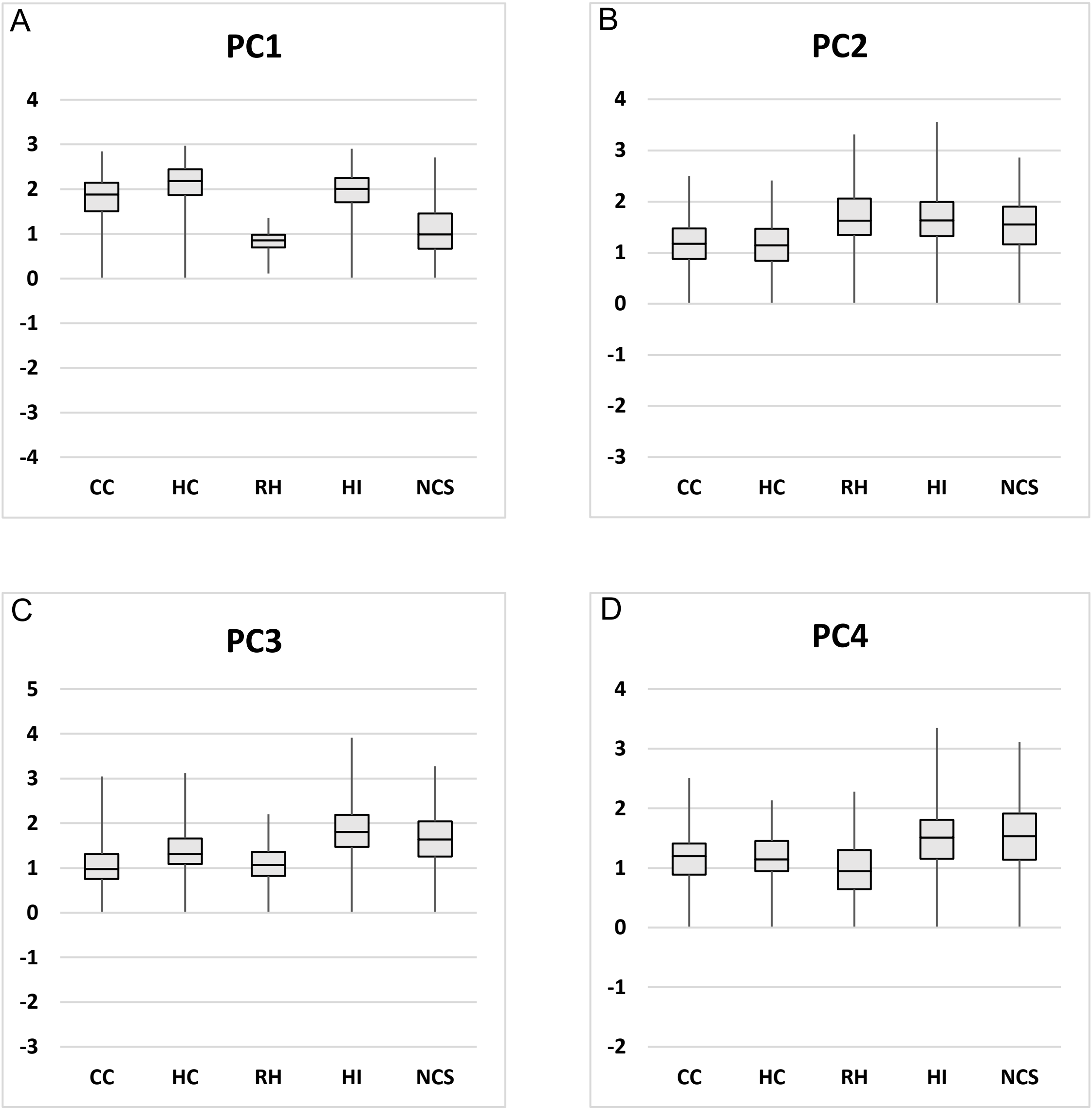

Supplementary Figure 2. Box plots represent the distribution of principal components (PCs) 1 - 4 (A-D). The center line in each box represents the median value. The top box represents the first quartile of the data and the bottom box represents the third quartile of the data. The top and bottom whiskers represent the maximum and minimum values (respectively) in the dataset. CC: *V. cinerea* B9 by 'Chardonnay;' HC: 'Horizon' by *V. cinerea* B9; RH: *V. rupestris* B38 by 'Horizon;' HI: 'Horizon' by Illinois 547-1; and NCS: 'Norton' by 'Cabernet Sauvignon.'
