# Supplementary material for: Quantitative Trait Locus Analysis of Leaf Morphology Indicates Conserved Shape Loci in Grapevine
Source: Front Plant Sci. 2019 Nov 15;10:1373. doi: 10.3389/fpls.2019.01373 (PMC6873345; doi:10.3389/fpls.2019.01373)
Supplement: Supplementary file 3 [file Image_3.pdf]

A

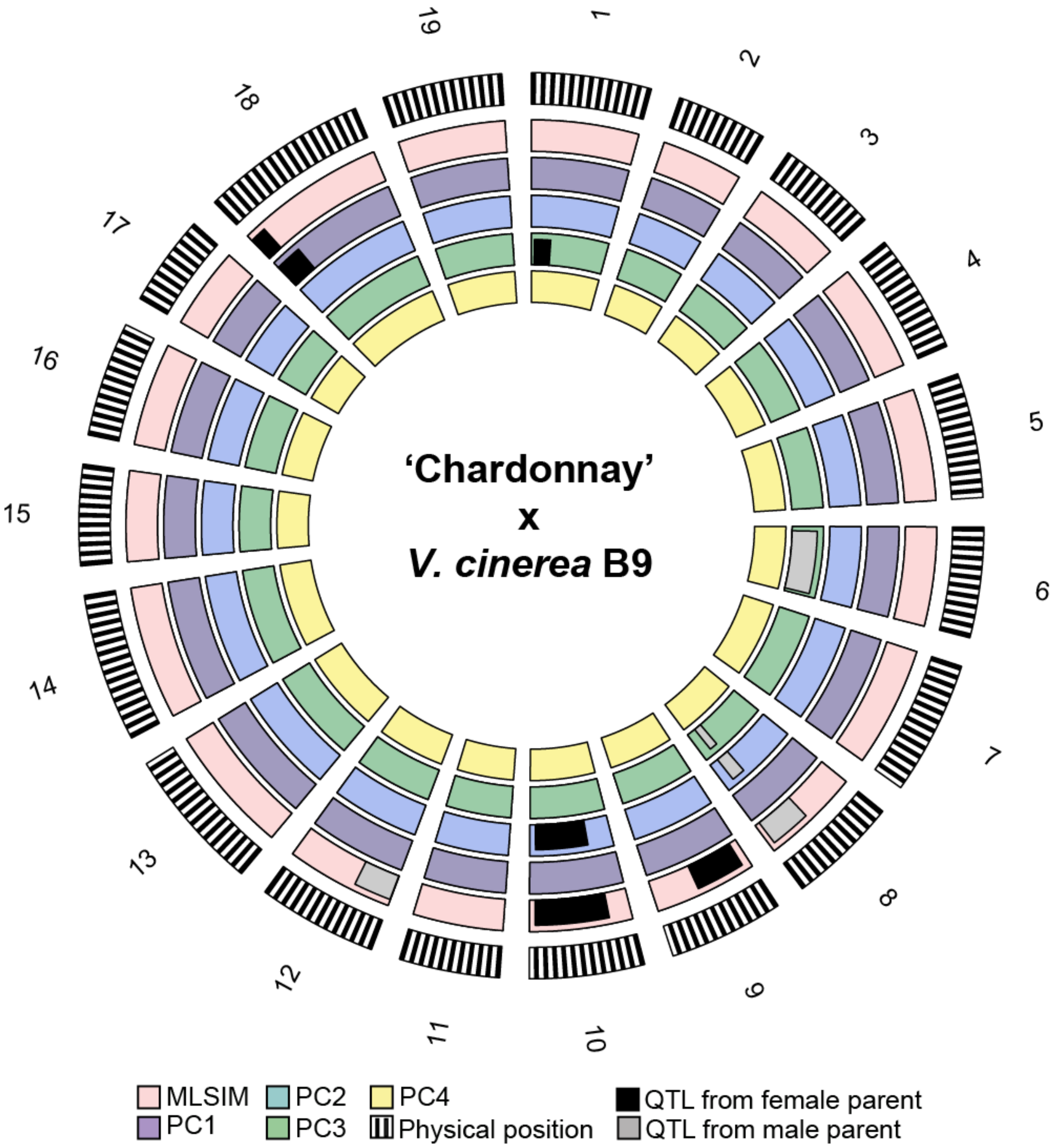
'Chardonnay'
x
V. cinerea B9
1
2
3
4
5
6
7
8
9
10
11
12
13
14
15
16
17
18
19
MLSIM
PC1
PC2
PC3
PC4
Physical position
QTL from female parent
QTL from male parent

B

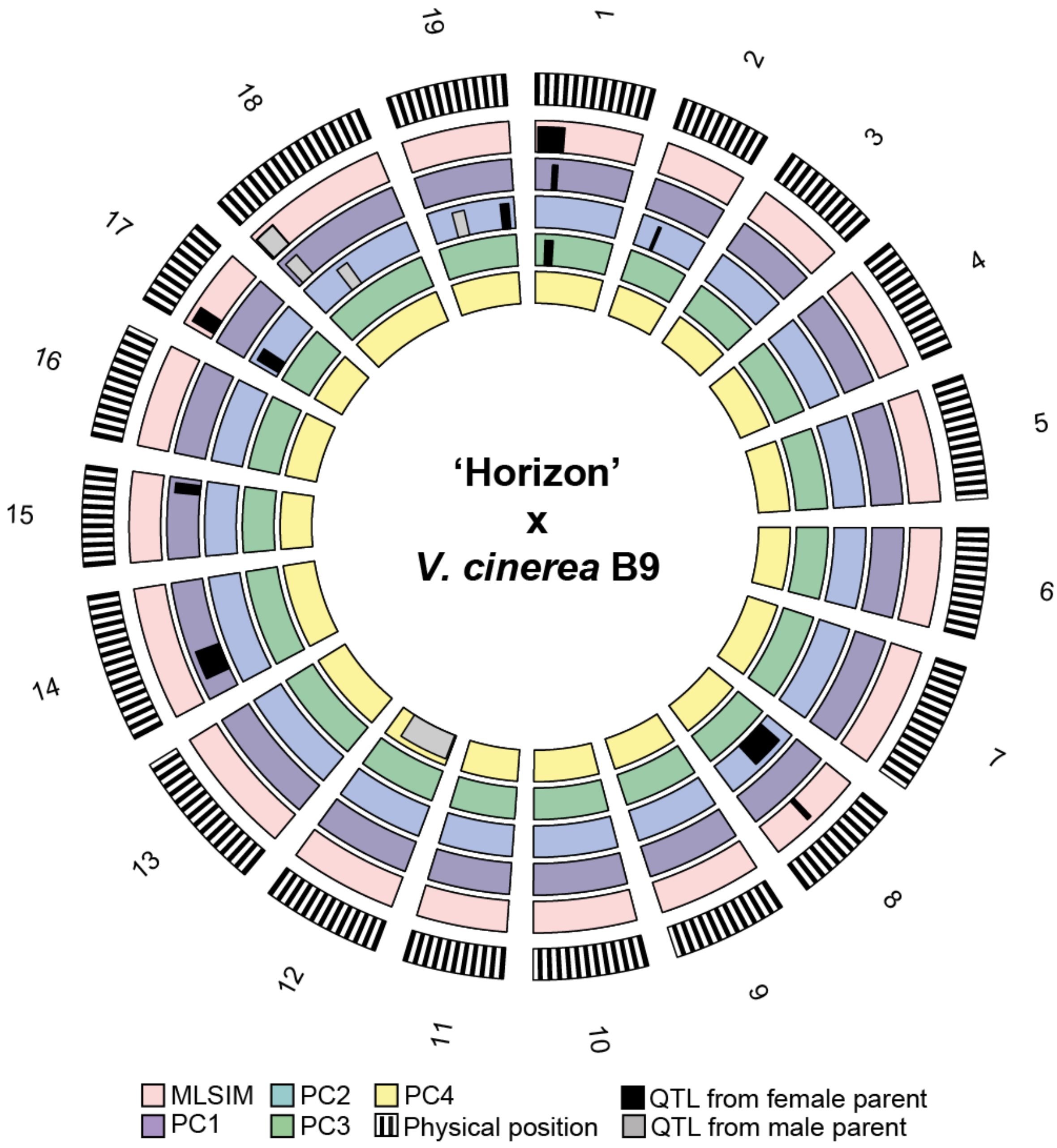

1
2
3
4
5
6
7
8
9
10
11
12
13
14
15
16
17
18
19
'Horizon'
x
V. cinerea B9
MLSIM
PC1
PC2
PC3
PC4
Physical position
QTL from female parent
QTL from male parent

C

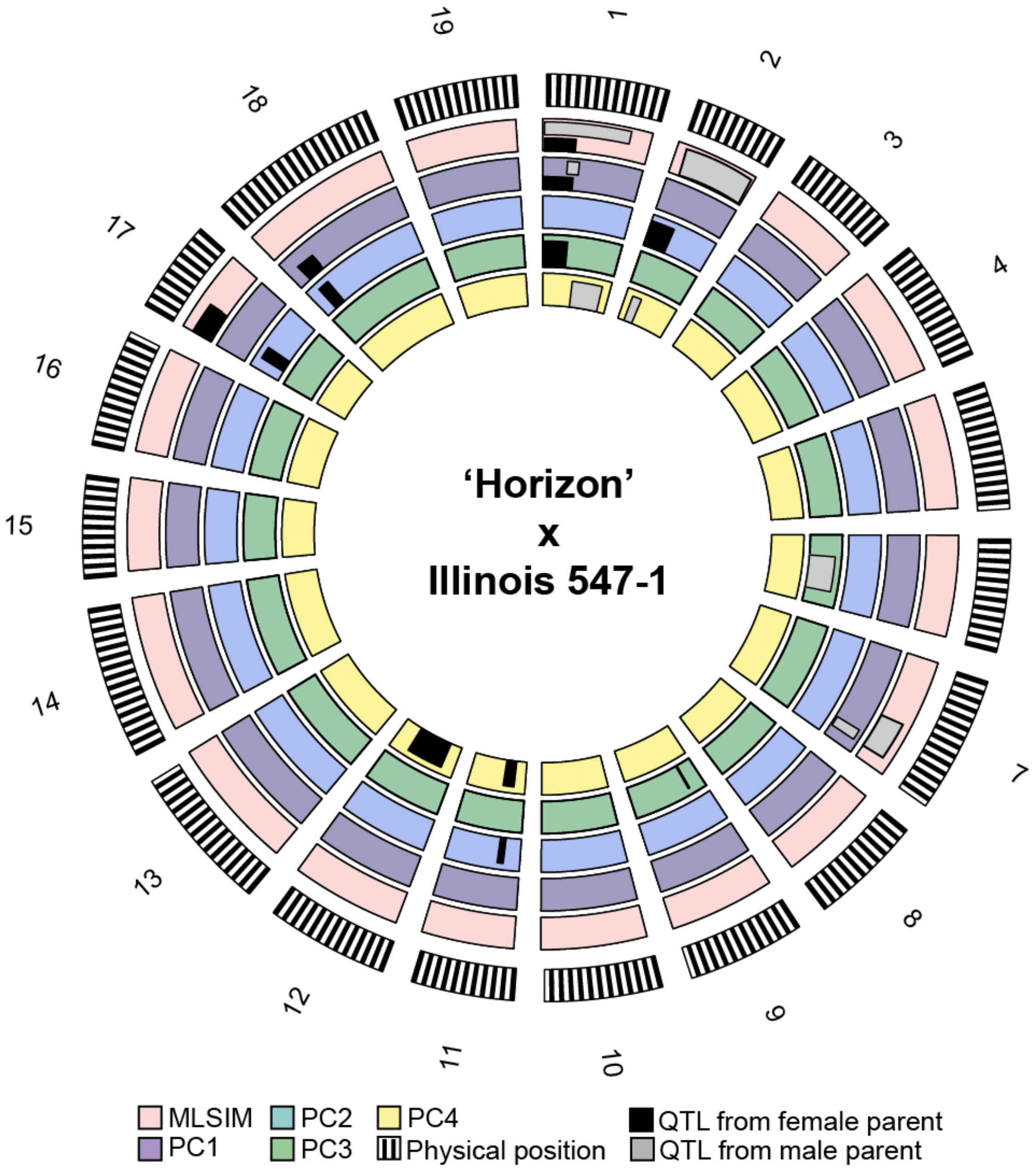

'Horizon'
x
Illinois 547-1
1
2
3
4
5
6
7
8
9
10
11
12
13
14
15
16
17
18
19
MLSIM
PC1
PC2
PC3
PC4
Physical position
QTL from female parent
QTL from male parent

D

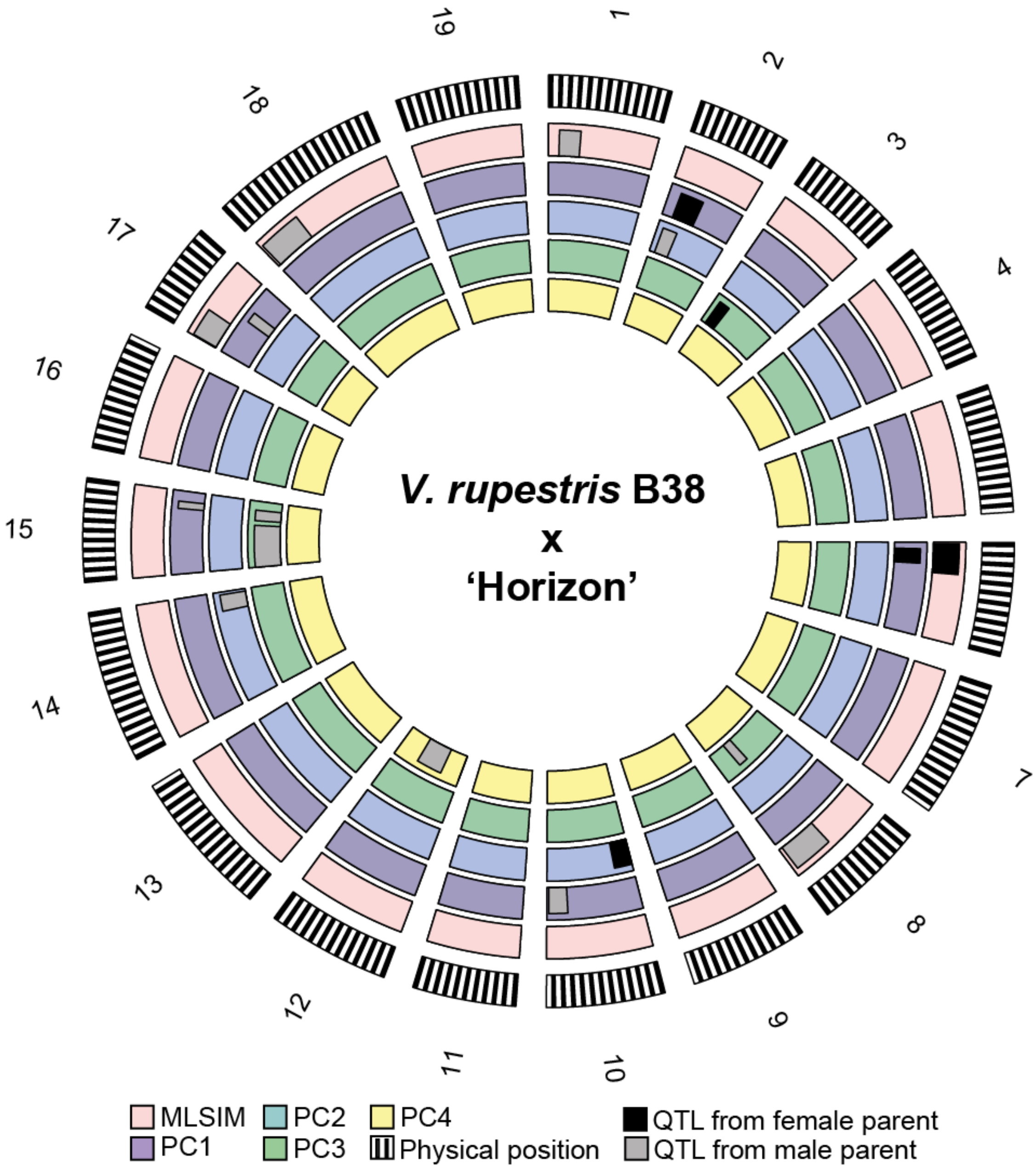
V. rupestris B38
x
'Horizon'
1
2
3
4
5
6
7
8
9
10
11
12
13
14
15
16
17
18
19
MLSIM
PC1
PC2
PC3
PC4
Physical position
QTL from female parent
QTL from male parent

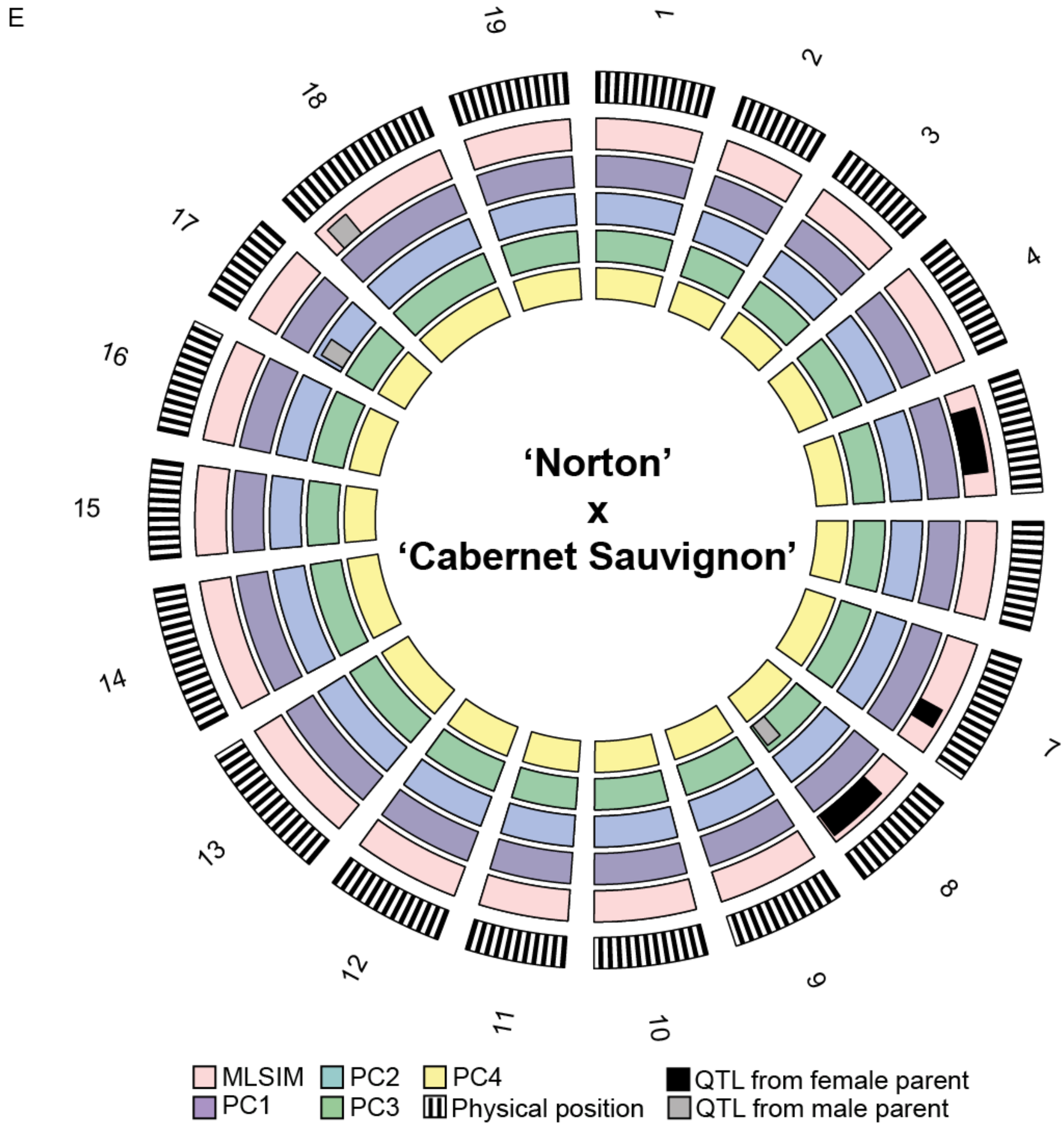

Supplementary Figure 3. Circular plots for each of the five mapping families illustrate genomic regions identified from the PC QTL analyses: A. *V. cinerea* B9 by 'Chardonnay;' B. 'Horizon' by *V. cinerea* B9; C. 'Horizon' by Illinois 547-1; D. *V. rupestris* B38 by 'Horizon;' and E. 'Norton' by 'Cabernet Sauvignon'. Chromosomes 1 through 19 are labeled. The physical distance of each chromosome is represented as the outer circle. Each alternating black and white bar on the outer circle represents 1 Mbp. The remaining 5 concentric circles represent QTL identified from the PC analyses. QTL identified are depicted as bars spanning the distance of each interval. Black bars represent QTL from the female parent and gray bars represent QTL from the male parent.
