# Supplementary material for: Quantitative Trait Locus Analysis of Leaf Morphology Indicates Conserved Shape Loci in Grapevine
Source: Front Plant Sci. 2019 Nov 15;10:1373. doi: 10.3389/fpls.2019.01373 (PMC6873345; doi:10.3389/fpls.2019.01373)
Supplement: Supplementary file 4 [file Image_4.pdf]

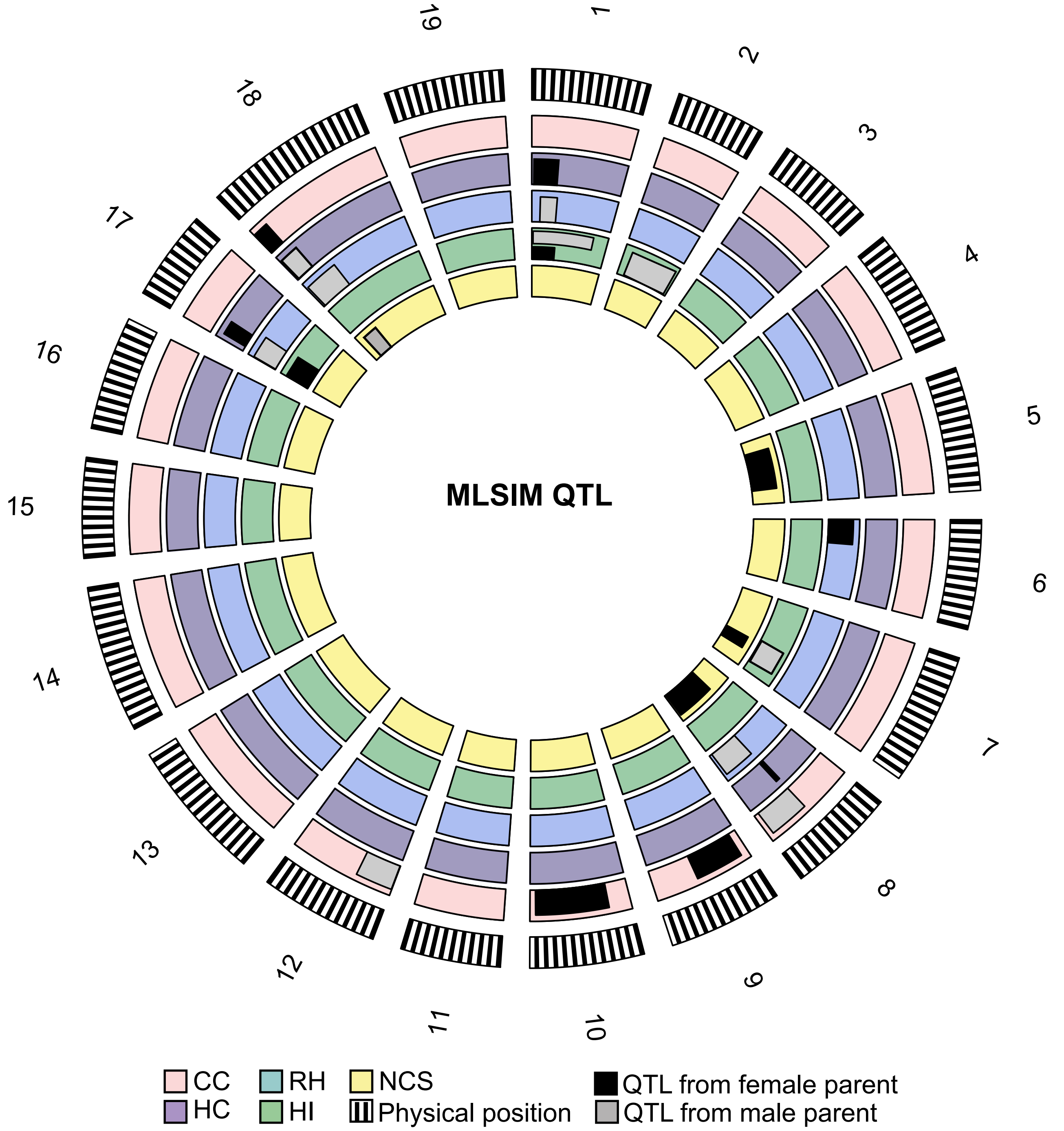

Supplementary Figure 4. Side-by-side comparison of significant QTL obtained from MLSIM. Circular plot illustrates overlapping genomic regions between the mapping families: *V. cinerea* B9 × 'Chardonnay'; 'Horizon' × *V. cinerea* B9; 'Horizon' × Illinois 547-1; *V. rupestris* B38 × 'Horizon;' and 'Norton' × 'Cabernet Sauvignon.' Intervals between the Wilks' upstream and downstream limits for QTL identified using MLSIM with greater than 5% phenotypic variance and LOD intervals of QTL identified are plotted by physical position using the 12X.v2 PN40024 *V. vinifera* reference genome. Chromosomes 1 through 19 are labeled. The physical distance of each chromosome is represented as the outer circle. Each alternating black and white bar on the outer circle represents 1 Mbp. QTL identified are depicted as bars spanning the distance of each interval. Black bars represent QTL from the female parent and gray bars represent QTL from the male parent.
